# Traditional Chinese medicine Lianhua Qingwen for treating COVID-19: A protocol for a meta-analysis and systematic review of randomized controlled trials

**DOI:** 10.1097/MD.0000000000024204

**Published:** 2021-01-15

**Authors:** Shasha Li, Jingxia Zhang, Fan Li, Ajuan Mao, Yajuan Li, Chongbo Zhao, Xiaowei Hu, Fang Li, Weifeng Wang

**Affiliations:** a Shaanxi Academy of Traditional Chinese Medicine, Xi’an, Shaanxi, China; b Pharmacy College, Shaanxi University of Chinese Medicine, Xianyang; c Shaanxi Traditional Chinese Medicine Hospital, Xi’an, Shaanxi, China.

**Keywords:** COVID-19, lianhua qingwen, meta-analysis, protocol, systematic review

## Abstract

**Background:**

Since the outbreak of coronavirus disease 2019 (COVID-19) in 2019, it has swept the world with rapid development and is one of the infectious diseases that seriously threatened global public health. Because of the complex pathogenesis, high infectivity, and high fatality rate of COVID-19, there are no effective treatments for this epidemic at present. Traditional Chinese Medicine (TCM) has a long clinical history in the prevention and treatment of such acute infectious diseases. The therapeutic effect of Lianhua Qingwen (LHQW) on this new coronary pneumonia has attracted the attention of all walks of life, and relevant research reports continue to appear. Here, we intend to conduct a systematic review and meta-analysis of randomized controlled trials (RCT) to evaluate the efficacy of LHQW in COVID-19 patients.

**Methods:**

We will search each database from the built-in until Dec 2020. The English literature mainly search the Cochrane Library, EMBASE, PubMed, and Web of Science, while the Chinese literature come from CNKI, VIP, Chinese Biomedical Database (CBM), Chinese Science Citation Database (CSCD), and Wan Fang database. Simultaneously, we will retrieve clinical registration tests. This study only screens the RCT of LHQW against COVID-19 and evaluates its efficacy and safety. We will use the Cochrane Handbook to systematically review interventions to assess the risk of bias. The protocol will be reported according to the approach and preferred report items for systematic review and meta-analysis protocols (PRISMA - P). Finally, RevMan software version 5.3 will be used for meta-analysis.

**Results:**

The systematic review and meta-analysis aim to review and pool current clinical outcomes of LHQW for treating COVID-19.

**Conclusion:**

This study will provide further evidence for the efficacy and safety of LHQW in the treatment of COVID-19.

**INPLASY Registration number:**

INPLASY2020120043.

## 1 Introduction

COVID-2019 is an acute infectious disease caused by the novel coronavirus type 2 acute respiratory syndrome coronavirus (SARS-CoV-2), which is the first coronavirus pandemic declared by WHO. It has caused more than 66.2 million infections and more than 1.5 million deaths. Among them, 93,797 cases of COVID-19 (including imported cases) were diagnosed in China, 87,677 cases were cured, and 4751 died.^[1]^ From these data, it is not difficult to find that China's prevention and control of the COVID-19 epidemic situation is applicable. It is not only because of measures taken by Chinese government at all levels, including preventing large public or private gatherings, wearing masks and gloves, and avoiding unnecessary travel and visitation, but also depends on the important factors of medical assistance.^[1–3]^ In the pandemic, the Chinese government has provided valuable reference for countries around the world in epidemiology, diagnosis, and management, especially the TCM has played a huge role in the prevention and control of COVID-19.^[4]^

After the outbreak of the coronavirus disease, the National Health Commission of the People's Republic of China has formulated and issued “the Diagnosis and Treatment Protocol for Novel Coronavirus Pneumonia (Trial Version)” based on the clinical manifestations, pathological of the disease and the experience of diagnosis and treatment of SARS in 2003.^[5]^ Chinese herbal medicine, a medical system with regional characteristics, was incorporated into management for COVID-19 in the early stage of the onset. LHQW appears in the protocol as one of the recommended Chinese medicine prescriptions, which comprises ancient prescription Yin Qiao San and Ma Xing Shi Gan Decoction. It formula was composed of 13 herb components: Forsythia Fructus, Lonicerae Japonicae Flos, Ephedrae Herba, Armeniacae Semen Amarum, Gypsum Fibrosum, Isatidis Radix, Dryopteridis Crassirhizomatis Rhizoma, L-menthol, Houttuyniae Herba, Pogostemonis Herba, Rhei Radix et Rhizoma, Rhodiolae Crenulatae et Rhizoma, and Glycyrrhizae Radix et Rhizoma. Common formulation of this formula are capsule, granule, and decoction.^[6]^ In terms of modern pharmacological analysis, the prescription has multiple functions of antiviral, anti-inflammatory, antibacterial, antipyretic, and so on.^[7]^ It could inhibit SARS-CoV-2 replication, alter the viral morphology, and reduce the cytokine release from host cells.^[8]^ LHQW has been used for fighting against the COVID-19 that it was clinically shown to have a beneficial effect.^[9]^ Therefore, this study intends to collect RCT of LHQW for COVID-19, which is based on evidence-based medicine, and conduct a meta-analysis of its efficacy and safety to provide higher quality clinical evidence for Chinese medicine treatment of COVID-19.

## 2 Methods

### 2.1 Protocol registration

The systematic review protocol has been registered on the International Platform of Registered Systematic Review and Meta-Analysis Protocols (INPLASY). The registration number was INPLASY2020120043. The protocol is reported following the guidelines on the Cochrane Handbook for Systematic Reviews of Interventions and the PRISM-P.^[10]^ We will update our protocol for any changes in the entire research process if needed.

### 2.2 Inclusion criteria

#### 2.2.1 Study design

The study only selects clinical randomized controlled trials of LHQW for COVID-19 published in both Chinese and English.

#### 2.2.2 Participants

This study included patients who had been clearly diagnosed with COVID-19. Except that participants must be over 18 years old, there were no strict restrictions on gender, racial group, and severity of the disease.

#### 2.2.3 Intervention

The test group was given LHQW combined with conventional treatment. The control group was only given conventional treatment. There are no obvious restrictions on the dosage of therapeutic drugs and specific intervention routes.

#### 2.2.4 Types of outcome measures

The main outcomes include total effective rate, relief time of major symptoms (such as fever, cough, sore throat), Secondary outcomes include relief time of other symptoms (such as headache, dizziness, diarrhea, nausea and so on), lung CT, adverse events.

### 2.3 Search methods

We will retrieve each database until Dec 2020. English literature mainly searches the Cochrane Library, PubMed, EMBASE, and Web of Science, While Chinese literature comes from CNKI, VIP, CBM, CSCD, and Wan Fang database. Chinese Clinical Trial Registry and Clinical Trials.gov will also be searched to discover ongoing trials with unpublished data, and the Conference abstracts will be searched manually. We adopt the combination of heading terms and free words as a search strategy that is decided by all the reviewers. We will simply provide the search process of the PubMed (Table 1). We will adjust diverse search methods according to different Chinese and English databases.

**Table 1:** PUBMED search strategy.

Number	Search terms
#1	Mesh descriptor: (Lianhua Qingwen) explode all trees
#2	((((Lianhua Qingwen [Title/Abstract])) OR (Lian Hua Qing Wen [Title/Abstract])) OR ((lianhuaqingwen [Title/Abstract])) OR (LIan-Hua Qing-Wen [Title/Abstract])
#3	Or #1–#2
#4	Mesh descriptor: (COVID-19) explode all trees
#5	((((((((COVID-19 [Title/Abstract])) OR (2019 novel coronavirus disease [Title/Abstract])) OR (coronavirus disease 2019 [Title/Abstract])) OR (2019 novel coronavirus infection [Title/Abstract])) OR (COVID-19 pandemic [Title/Abstract])) OR (COVID-19 virus infection [Title/Abstract])) OR (SARS-CoV-2 infection [Title/Abstract])) OR (2019-nCoV infection [Title/Abstract])
#6	Or #4–#5
#7	Mesh descriptor: (randomized controlled trials) explode all trees
#8	((((((random [Title/Abstract])) OR (randomly [Title/Abstract])) OR (random allocation [Title/Abstract])) OR (clinical trials [Title/Abstract])) OR (randomized control trial [Title/Abstract])) OR (controlled clinical trials [Title/Abstract])
#9	Or #7–#8
#10	#3 and #6 and #9

### 2.4 Data collections and analysis

#### 2.4.1 Selection of studies

**Figure 1:**
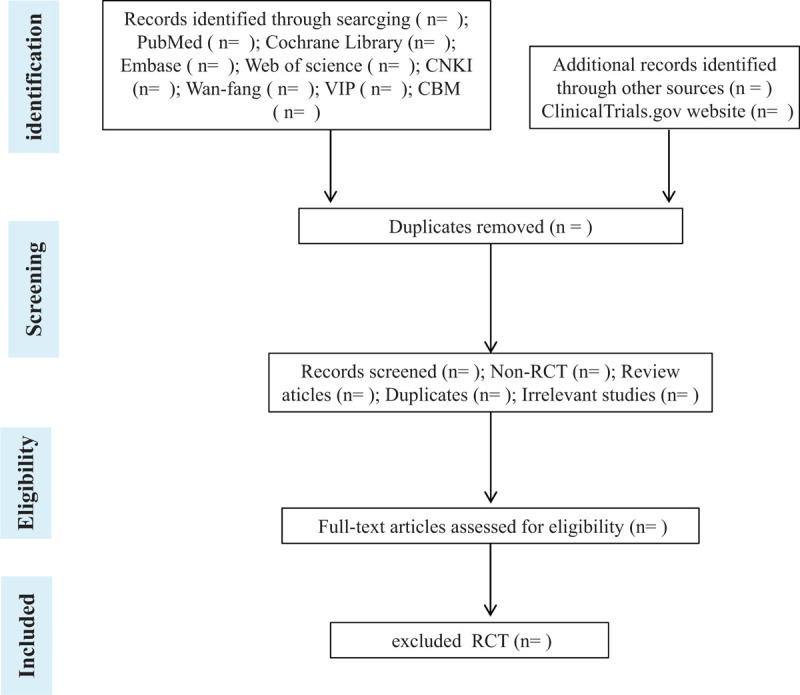
Process of study screening.

Import all literature that meets the criteria into Endnote X8 software. First, 2 independent reviewers initially screened the literature that did not meet the pre-established standards of the study by reading the title and abstract. Second, download the remaining literature and read the full text carefully to further decide whether or not to. Finally, the results were cross-checked repeatedly by reviewers. If there is a disagreement in the above process, we can achieve agreement by discussing both reviewers or seek a third partys opinion. All screening and managing processes of the articles will be performed with Endnote software. Flow chart of the study selection (Fig. 1) will be used to show the screening process of the study.

#### 2.4.2 Data extraction and management

According to the characteristics of the study, 2 independent reviewers will extract the main data with an excel file. The main data extracted are follows: Title, author, year, fund source, country, language, journal source, study design, sample size, age, interventions, the type of measures, adverse reactions, risk of bias assessment, etc. The above information was finally cross-checked by 2 reviewers, and any disagreements will be resolved by consensus, with any ongoing differences in perspective being arbitrated by a third reviewer.

#### 2.4.3 Assessment of risk of bias in included studies

The quality of included studies was assessed by 2 independent reviewers according to the Cochrane Handbook for Systematic Reviews of interventions. The following 7 items, such as random sequence generation (selection bias), allocation concealment (selection bias), blinding of participants and personnel (performance bias), blinding of outcome assessment (detection bias), incomplete outcome data (attrition bias), selective outcome reporting (reporting bias) and other bias, are evaluated by 3 grades of “low bias”, “high bias”, and “unclear bias”. The discrepancies will get a consistent conclusion by discussing both reviewers or seeking the third reviewer consultation.

#### 2.4.4 Data analysis

Meta-analyses will be conducted when at least 2 studies are included. We will conduct data synthesis and analysis using the RevMan software (version 5.3.5). If over 10 studies are included, a funnel plot will be used to assess reporting bias, and if less than 10studies are included, *P* value will be used. In the process, 2 investigators will extract detailed information and available data from the qualified studies, and select different evaluation methods according to the different efficacy indicators. The effect scale indicator relative risk and 95% confidence interval (CI) be used to investigate dichotomous data. For continuous data, we will choose the mean difference or standardized mean difference with 95% CI to represent, this depending on the measurement scale is consistent or not. If there is no heterogeneity (*I*^2^ < 50%, *P* > .1), the data are synthesized using a fixed-effect model. Otherwise (*I*^2^ ≥ 50%, *P* < .1), a random effect model is used to analyze. Then subgroup analysis will be conducted basing on the diverse causes of heterogeneity. If a meta-analysis can not be performed, it will be replaced by a general descriptive analysis.

## 3 Discussion

COVID-19 is an acute infectious respiratory disease caused by novel coronavirus type 2 acute respiratory syndrome coronavirus. It can result in fever, dry cough, fatigue, multiple organ dysfunction, and death. Currently, there are no effective drugs to treat patients with COVID-19, The TCM plays an role in dealing with the impact of the disease on the respiratory system.

LHQW could reduce patient mortality, improve clinical symptoms (such as fever, cough, fatigue) for COVID-19.^[11]^ The results network pharmacology showed that LHQW prescription acted on coronavirus through multiple components, multiple targets, and multiple pathways. The major components not only have good binding ability with ACE2, but also effectively bind to the contact surface of ACE2 and the spinous process complex, thus playing a potential therapeutic role in COVID-19.^[12,13]^ Therefore, this study intends to evaluate the safety and safety efficacy of LHQW in COVID-19 patients through systematic reviews and meta-analysis.

## Author contributions

**Methodology:** Shasha Li.

**Project administration:** Shasha Li.

**Software:** Jingxia Zhang, Ajuan Mao, Yajuan Li.

**Supervision:** Fan Li.

**Visualization:** Chongbo Zhao, Xiaowei Hu.

**Writing – original draft:** Shasha Li, Jingxia Zhang.

**Writing – review & editing:** Fang Li, Weifeng Wang.

## Abbreviations

Abbreviations: CI = confidence interval, COVID-19 = corona virus disease 2019, LHQW= lianhua qingwen, PRISMA - P = Preferred Reporting Items for Systematic Reviews and Meta-Analysis Protocol, RCT = randomized controlled trial, TCM = traditional Chinese medicine.
